# Subshifts on Infinite Alphabets and Their Entropy

**DOI:** 10.3390/e22111293

**Published:** 2020-11-13

**Authors:** Sharwin Rezagholi

**Affiliations:** Max Planck Institute for Mathematics in the Sciences, Inselstrasse 22, 04103 Leipzig, Germany; sharwin.rezagholi@mis.mpg.de

**Keywords:** infinite graphs, symbolic dynamics, topological entropy, word complexity

## Abstract

We analyze symbolic dynamics to infinite alphabets by endowing the alphabet with the cofinite topology. The topological entropy is shown to be equal to the supremum of the growth rate of the complexity function with respect to finite subalphabets. For the case of topological Markov chains induced by countably infinite graphs, our approach yields the same entropy as the approach of Gurevich We give formulae for the entropy of countable topological Markov chains in terms of the spectral radius in $l^{2}$.

## 1. Introduction

Symbolic dynamical systems on finite alphabets are classical mathematical objects that provide a wealth of examples and have greatly influenced theoretical developments in dynamical systems. In computer science, certain symbolic systems, namely, the topological Markov chains generated by finite graphs, model the evolution of finite transition systems, and the class of sofic symbolic systems (factors of topological Markov chains) models the evolution of certain automata. The most important numerical invariant of dynamical systems is the topological entropy. For symbolic systems, the entropy equals the exponential growth rate of the number of finite words of fixed length. In the case of a topological Markov chain, the entropy equals the natural logarithm of the spectral radius of the generating graph. Considering the graph as a linear map, the spectral radius measures the rate of dilation under iterated application. On an exponential scale, this rate equals the growth of the number of finite words. This note is an attempt to generalize this meeting ground of topology, graph theory, and spectral theory to infinite alphabets, especially countably infinite alphabets. Beside its theoretical interest, this was motivated by the increasing importance of infinite state systems in computer science.

Any attempt at studying symbolic dynamics on infinite alphabets has to deal with the fact that the discrete topology, employed in the finite case, leads to shift spaces which are not compact. Most approaches attempt to compactify the respective alphabet. In this note, we endow the alphabet with a compact topology which coincides with the discrete topology in the finite case, the cofinite topology. Gurevich [1] has considered the Alexandrov compactification of the alphabet and his formula for the entropy of the respective topological Markov chains coincides with ours. The theory of Gurevich has the unpleasent feature that the closure of the set of graph walks must be taken. Still, our approach is similar to Gurevich’s, since minimal open covers in the Alexandrov compactification of an infinite countable discrete space and in the cofinite topology are similar. In Gurevich’s setting, the dynamical properties of the boundary of a (sofic) subshift have been studied ([2] Section 3) (see also [3,4,5]). Another approach is due to Ott et al. [6], who considered an N-shift on words in the Alexandrov compactification which they endowed with a certain quotient topology to get rid of the introduced *∞*-symbol. Their constructions have been further developed to yield topological dynamical systems which are analogous to classical Z-shifts, and in this setting, characterizations of morphisms of systems are known [7,8]. Contrary to these authors, this paper is mostly interested in entropy theory, especially its connections to subword complexity and spectral theory.

The entropy theory of this note admits a clear operational interpretation. In the case of an infinite alphabet, the number of finite words may be uncountable; hence, we identify all but finitely many letters prior to counting. The entropy is obtained by suprematizing over such identifications. Under some conditions, the entropy of a countable topological Markov chain may be computed or bounded via the spectral radius of a linear operator, analogous to the finite case. This reduces the computation of the entropy of certain symbolic systems on countable alphabets to a well-understood numerical problem.

Section 2 recalls some of the theory of symbolic dynamics on finite alphabets, Section 3 defines subshifts on infinite alphabets, Section 4 shows that the entropy equals the supremum of the exponential growth rates of the number of words in a finite subalphabet, Section 5 specializes to the case of countably infinite topological Markov chains and provides spectral formulae, Section 6 presents a proof that all nonnegative real numbers may be the entropy of a subshift on an infinite alphabet, and Section 7 collects examples.

## 2. Symbolic Dynamics on Finite Alphabets

We recall the construction of symbolic dynamical systems on a finite alphabet. Consider a finite set of letters, the alphabet, {1,…,k}=:[k], endowed with the discrete topology. We form the product space ${\left[k\right]}^{Z}$ on which the shift map $\sigma :{\left[k\right]}^{Z}\to {\left[k\right]}^{Z}$ acts continuously via $\sigma {\left(s\right)}_{i}=s_{i+1}$. The dynamical system $\left({\left[k\right]}^{Z},\sigma \right)$ is called the *k*-shift. A subshift of the *k*-shift is a closed σ-invariant subset $S\subseteq {\left[k\right]}^{Z}$; we write (S,σ). Special cases of subshifts are the topological Markov chains; they are induced by finite directed graphs. We treat such graphs as finite square matrices with entries from the set {0,1}, the respective adjacency matrices. Given a graph *G*, whose vertex set we may enumerate as [k], its associated subshift is $\left(S_{G},\sigma \right)$ where

$$S_{G}:=\left\{s\in {\left[k\right]}^{Z}|G_{s_{t}s_{t+1}}=1\;\mathrm{for}\;\mathrm{all}\;t\in Z\right\}.$$

We denote by $W^{t}\left(S\right)$ the words of length *t* in the subshift *S*; hence

$$W^{t}\left(S\right):=\left\{\{a_{1},...,a_{t}\}|\exists s\in S\;\mathrm{with}\;\left\{s_{i},...,s_{i+t-1}\right\}=\left\{a_{1},...,a_{t}\right\}\;\mathrm{for}\;\mathrm{some}\;i\in Z\right\}.$$

The growth rate of a real nonnegative sequence ${\left\{x_{t}\right\}}_{t=1}^{\infty }$ is the expression

$${GR}_{t}\left(x_{t}\right):=\underset{t\to \infty }{lim sup}\frac{1}{t}\ln^{+}\left(x_{t}\right)$$

where $\ln^{+}\left(x\right):=\max\left\{0,\ln\left(x\right)\right\}$. The above is the asymptotic exponential growth rate of the sequence. It may be zero, if the sequence grows subexponentially, or infinite, if the sequence grows superexponentially. Given a subshift *S*, its complexity function assigns $t\mapsto |W^{t}\left(S\right)|$. The growth rate ${GR}_{t}\left(|W^{t}\left(S\right)|\right)$ measures the asymptotic exponential growth rate of the number of words in the subshift. In the case of a topological Markov chain, one has [9]

$${GR}_{t}\left(|W^{t}\left(S_{G}\right)|\right)=\ln^{+}\left(\lambda_{G}^{\max}\right)$$

where $\lambda_{G}^{\max}$ denotes the largest eigenvalue of the graph *G*.

The topological entropy [10] is the chief numerical invariant associated with a topological dynamical system. Let *X* be a compact topological space and let f:X→X be continuous. The topological entropy of the system (X,f) with respect to the open cover U of *X* is

$$h_{U}\left(X,f\right)={GR}_{t}\left(\#\vee_{i=0}^{t}f^{-i}U\right)$$

where $A\vee B:={\left\{A\cap B\right\}}_{A\in A,B\in B}$, and #C denotes the minimal cardinality of a finite subcover of C. The topological entropy of the system (X,f) is

$$h\left(X,f\right)=\sup\left\{h_{U}\left(X,f\right)|\;U\;\mathrm{is}\;\mathrm{an}\;\mathrm{open}\;\mathrm{cover}\;\mathrm{of}\;X\right\}.$$

The entropy of a subsystem is lesser or equal to the entropy of the surrounding system. The entropy of the continuous image of a system is lesser or equal to the original entropy. It is well-known [10] that for a subshift (S,σ) we have $h\left(S,\sigma \right)={GR}_{t}\left(|W^{t}\left(S\right)|\right)$. In particular, $h\left(S_{G},\sigma \right)=\ln^{+}\left(\lambda_{G}^{\max}\right)$.

## 3. Symbolic Dynamics on Infinite Alphabets

Let *A* be a set equipped with the cofinite topology, the minimal topology with the $T_{1}$-property, which is generated by the subbasis ${\left\{A\backslash \{x\}\right\}}_{x\in A}$. The respective basis consists of sets of the form $A\backslash \{x_{1},\ldots ,x_{n}\}$. It is easy to verify that this basis is the entire topology. Hence, a subset of *A* is open if and only if it is the complement of a finite subset. If *A* is finite, the cofinite topology coincides with the discrete topology. The cofinite topology turns every set into a compact separable $T_{1}$-space. If *A* is infinite, its cofinite topology is hyperconnected. By Tikhonov’s theorem, the product $A^{Z}$ is also compact. The topology of $A^{Z}$ has a subbasis of sets of the form

$$U\left(t,x\right):=\left\{s\in A^{Z}|s_{t}\in A\backslash \left\{x\right\}\right\}=\left\{s\in A^{Z}|s_{t}\neq x\right\}$$

where t∈Z and x∈A. The shift map $\sigma :A^{Z}\to A^{Z}$ defined by $\sigma {\left(s\right)}_{t}=s_{t+1}$ is continuous since

$$\sigma^{-1}\left(U(t,x)\right)=U\left(t-1,x\right).$$

A basis for $A^{Z}$ is given by finite intersections of subbasic open sets, by sets of the form

$$\left\{s\in A^{Z}|s_{t_{1}}\neq x_{1},\ldots ,s_{t_{n}}\neq x_{n}\right\}.$$

In the remainder of this note, we suppose shift spaces to be equipped with the product of the cofinite topology, unless we explicitly state otherwise.

**Definition** **1** (Shifts and subshifts). *The shift is the dynamical system $\left(A^{Z},\sigma \right)$ for some alphabet A. A subshift of $\left(A^{Z},\sigma \right)$ is a dynamical system (S,σ) where $S\subseteq A^{Z}$ is closed and σ(S)⊆S.*

By a morphism from the subshift $S\subseteq A^{Z}$ to the subshift $T\subseteq B^{Z}$ we mean a σ-equivariant continuous surjection M:S→T. The following diagram commutes.

$$\begin{array}{ccc} S & \overset{\sigma }{⟶} & S\\ ↓M & & ↓M\\ T & \overset{\sigma }{⟶} & T \end{array}$$

If *S* has an infinite alphabet, codings may not lead to morphisms, as the following proposition shows (See also ).

**Proposition** **1** (Sliding block codes). *Let $S\subseteq A^{Z}$ be a subshift. Consider a map $m:A^{\left\{-t,...,t\right\}}\to B$ and define the σ-equivariant map $M:A^{Z}\to B^{Z}$ by $M{\left(s\right)}_{i}=m\left(\left\{s_{i-t},...,s_{i+t}\right\}\right)$. Then M:S→M(S) is continuous if $|m^{-1}\left(b\right)\cap W^{2t+1}\left(S\right)|<\infty$ for all b∈B.*

**Proof.**  Clearly M(S) is σ-equivariant. By pulling back a subbasic open set through *M*, we see

$$\begin{array}{ccc} M^{-1}\left(\left\{t\in M\left(S\right)|t_{i}\neq b\right\}\right) & = & \left\{s\in S|m(\left\{s_{i-t},...,s_{i+t}\right\})\neq b\right\}\\ & = & \left\{s\in S|\left\{s_{i-t},...,s_{i+t}\right\}\notin m^{-1}\left(b\right)\right\}\\ & = & \underset{w\in m^{-1}\left(b\right)}{⋂}\left\{s\in S|\{s_{i-t},...,s_{i+t}\}\neq w\right\}\\ & = & \underset{w\in m^{-1}\left(b\right)\cap W^{2t+1}\left(S\right)}{⋂}\left\{s\in A^{Z}|\left\{s_{i-t},...,s_{i+t}\right\}\neq w\right\}. \end{array}$$

where the latter is open if the intersection is finite. □

If *A* is finite, $m^{-1}\left(b\right)$ is finite, and therefore all sliding block codes give rise to morphisms. In fact, every morphism between subshifts on finite alphabets is a sliding block code, the Curtis–Hedlund–Lyndon theorem [11].

## 4. Entropy Theory

In the following, we restrict our attention to countably infinite alphabets. We may suppose, without loss of generality, that A=N. We denote by $W_{F}^{t}\left(S\right)$ the set of words of length *t* in the subshift *S* whose letters are from the finite subalphabet *F*.

**Lemma** **1.** *Let S be a subshift of $N^{Z}$. The cover of $N^{Z}$ given as*

$$U\left(n\right):={\left\{s\in N^{Z}|s_{0}=i\;\mathrm{or}\;s_{0}>n\right\}}_{i=1}^{n}$$

*is open and fulfills*

$$h_{U(n)}\left(S,\sigma \right)={GR}_{t}\left(|W_{\left[n\right]}^{t}|\right).$$

**Proof.**  The cover U(n) consists of open sets since

$$\left\{s\in N^{Z}|s_{0}=i\;\mathrm{or}\;s_{0}>n\right\}=\left\{s\in N^{Z}|s_{0}\in N\backslash F\right\}.$$

where F:={1,...,n}\{i} is a finite set. The elements of $\vee_{k=0}^{t}\sigma^{-k}\;U\left(n\right)$ are of the form

$$\begin{array}{c} \{s|\left\{s_{-t},...,s_{0}\right\}\;\mathrm{is}\;\mathrm{in}\;W_{\left[n\right]}^{t+1}\;\mathrm{or}\;\mathrm{might}\;\mathrm{be}\;\mathrm{obtained}\;\mathrm{from}\;a\;\mathrm{word}\;\mathrm{in}\;W_{\left[n\right]}^{t+1}\\ \mathrm{by}\;\mathrm{changing}\;\mathrm{its}\;\mathrm{letters}\;\mathrm{to}\;\mathrm{letters}\;\mathrm{bigger}\;n\}. \end{array}$$

These covers are minimal, since the sets of the form

$${\left\{s\in N^{Z}|\left\{s_{-t},...,s_{0}\right\}=w\right\}}_{w\in W_{\left[n\right]}^{t+1}}$$

are properly contained in a single element of the cover. Hence

$$\#\vee_{k=0}^{t}\sigma^{-k}\;U\left(n\right)=|W_{\left[n\right]}^{t+1}|.$$

The claim follows by invoking Lemma 2. □

**Lemma** **2.** *Let S be a subshift of $N^{Z}$. Then*

$${GR}_{t}\left(|W_{\left[n\right]}^{t}|\right)={GR}_{t}\left(|W_{\left[n\right]}^{c+t}|\right)$$

*for all n∈N and c∈N.*

**Proof.**  Follows by taking growth rates with respect to *t* on

$$|W_{\left[n\right]}^{t}|\leq |W_{\left[n\right]}^{c+t}|\leq n^{c}\cdot |W_{\left[n\right]}^{t}|.\;□$$

So far, we have shown that $h\left(S,\sigma \right)\geq {GR}_{t}\left(\right|W_{F}^{t}\left|\right)$ for any finite subalphabet F⊆A. Hence, $h\left(S,\sigma \right)\geq \sup_{F⊊N}{GR}_{t}\left(\right|W_{F}^{t}\left|\right)$, where *F* is a finite set. We proceed to show that the reverse inequality also holds.

**Lemma** **3.** *Let S be a subshift of $N^{Z}$ and consider the following open cover of $N^{Z}$.*

$$B\left(n,t\right):=\vee_{i=-t}^{t}\sigma^{i}\;U\left(n\right)$$

*Then*

$$\lim_{n,t\to \infty }h_{B(n,t)}\left(S,\sigma \right)\geq h\left(S,\sigma \right).$$

**Proof.**  Note that $B\left(n^{\prime },t^{\prime }\right)≻B\left(n,t\right)$ whenever $n^{\prime }>n$ and $t^{\prime }>t$. The set N with its discrete topology is homeomorphic to a relatively discrete subset of the real line via the bijection that assigns n∈N to 1/n∈R. Its one-point Alexandrov-compactification adds the point 0∈R. Therefore, the Alexandrov compactification of the discrete space N is metrized by

$$d\left(i,j\right)=|\frac{1}{i}-\frac{1}{j}|=\frac{1}{ij}\left|i-j\right|.$$

The open subsets of Alex(N) are all subsets of N and sets of the form C⊔{∞} where C⊆N is cofinite. The product space $\mathrm{Alex}{\left(N\right)}^{Z}$ is metrized by

$$d\left(s,s^{\prime }\right)=\sum_{t\in Z}\frac{1}{2^{\left|t\right|}}d\left(s_{t},s_{t}^{\prime }\right)=\sum_{t\in Z}\frac{1}{2^{\left|t\right|}}\frac{1}{s_{t}s_{t}^{\prime }}\left|s_{t}-s_{t}^{\prime }\right|.$$

By definition of basis for a topology, the entropy is reached in covers by basic open subsets. A basis for $\mathrm{CoFin}{\left(N\right)}^{Z}$ is given by sets of the form

$$\left\{s\in N^{Z}|s_{t_{1}}\in C_{1},...,s_{t_{n}}\in C_{n}\right\}$$

for *n* cofinite sets $C_{i}\subseteq N$. The set

$$\left\{s\in N^{Z}|s_{t_{1}}\in C_{1}⊔\left\{\infty \right\},...,s_{t_{n}}\in C_{n}⊔\left\{\infty \right\}\right\}$$

is an open subset of $\mathrm{Alex}{\left(N\right)}^{Z}$. This procedure assigns to a basic open cover C with respect to $\mathrm{CoFin}{\left(N\right)}^{Z}$ the basic open cover $U^{\prime }$ with respect to $\mathrm{Alex}{\left(N\right)}^{Z}$. This map preserves the growth rate. Hence, the entropy with respect to the cofinite topology is smaller or equal to the entropy with respect to the Alexandrov compactification. The observation that

$$\mathrm{diam}\left(B^{\prime }\left(n,t\right)\right)\overset{n,t\to \infty }{\to }0$$

suffices to conclude that $h\left(S,\sigma \right)\leq \lim_{n,t\to \infty }h_{B(n,t)}\left(S,\sigma \right)$. □

**Lemma** **4.** *Let S be a subshift of $N^{Z}$ and consider the following open cover of $N^{Z}$.*

$$B\left(n,t\right):=\vee_{i=-t}^{t}\sigma^{i}\;U\left(n\right)$$

*Then*

$${GR}_{l}\left(\#\vee_{i=0}^{l}\sigma^{-i}B\left(n,t\right)\right)\leq {GR}_{l}\left(|W_{\left[n\right]}^{l}|\right).$$

*In particular,*

$$h_{B(n,t)}\left(S,\sigma \right)=h_{B(n,1)}\left(S,\sigma \right)=h_{U(n)}\left(S,\sigma \right).$$

**Proof.**  We have

$$\#\vee_{i=0}^{l}\sigma^{-i}B\left(n,t\right)\leq |W_{\left[n\right]}^{2t+1+l}|.$$

By Lemma 2, we conclude ${GR}_{l}\left(|W_{\left[n\right]}^{2t+1+l}|\right)={GR}_{l}\left(|W_{\left[n\right]}^{l}|\right)$. □

We are ready to prove the main result.

**Theorem** **1.** *Let $S\subseteq A^{Z}$ be a subshift on a countable alphabet. Then*

$$h\left(S,\sigma \right)=\sup\left\{{GR}_{t}\left(|W_{F}^{t}|\right)|F\;\mathrm{is}\;a\;\mathrm{finite}\;\mathrm{subset}\;\mathrm{of}\;A\right\}.$$

**Proof.**  Pick an arbitrary bijection N↔A. By Lemma 1 we have

$$\underset{n\in N}{\sup}h_{U(n)}\left(S,\sigma \right)\leq h\left(S,\sigma \right),$$

while by Lemmas 3 and 4 we have

$$h\left(S,\sigma \right)\leq \lim_{n,t\to \infty }h_{B(n,t)}\left(S,\sigma \right)=\underset{n\in N}{\sup}h_{U(n)}\left(S,\sigma \right).$$

Combining these inequalities and using Lemma 1 we obtain $h\left(S,\sigma \right)=\sup_{n\in N}{GR}_{t}\left(|W_{\left[n\right]}^{t}|\right)$. Since $|W_{F}^{t}\left|\leq \right|W_{F^{\prime }}^{t}|$ whenever $F\subseteq F^{\prime }$ and since every finite subset of N is included in some [n], we have

$$h\left(S,\sigma \right)=\underset{F⊊N}{\sup}{GR}_{t}\left(|W_{F}^{t}|\right).\;□$$

**Corollary** **1.** *Let A be a countably infinite alphabet. Then $h(A^{Z},\sigma )=\infty$.*

**Proof.**  From $|W_{F}^{t}{\left|=|F\right|}^{t}$ we conclude

$$h\left(A^{Z},\sigma \right)=\underset{F⊊A}{\sup}{GR}_{t}{\left(|F\right|}^{t})=\underset{F⊊A}{\sup}\ln\left(|F|\right)=\underset{n\in N}{\sup}\ln\left(n\right)=\infty .\;□$$

**Remark** **1.** *Let $S\subseteq A^{Z}$ be a subshift and let F⊆A be a finite subset of the alphabet such that for all s∈S where $s_{t}\in F$ for some t∈Z we have $s_{l}\in F$ for all l∈Z. Then $F^{Z}\cap S\subseteq S$ is clearly σ-invariant, and also closed, since $F^{Z}$ is the product of finite and therefore closed sets.*

The above observation allows us to build subhifts on infinite alphabets as “disjoint unions” of subshifts on finite alphabets.

**Example** **1.** *Let ${\left\{F_{i}\right\}}_{i\in N}$ be a sequence of subshifts on finite alphabets. Label the alphabet of $F_{1}$ by $\left\{1,...,f_{1}\right\}$, the alphabet of $F_{2}$ by $\left\{f_{1}+1,...,f_{2}\right\}$, and so on. We have obtained a subshift of $N^{Z}$ whose entropy equals $\sup_{i\in N}h\left(F_{i},\sigma \right)$.*

## 5. Shifts along Infinite (Directed) Graphs

In this section, we consider countably infinite graphs. For any such graph we assume, without loss of generality, that its vertex set is N.

**Proposition** **2.** *Let G be an infinite countable graph. Then the set*

$$S_{G}:=\left\{s\in N^{Z}|G_{s_{t},s_{t+1}}=1\;\mathrm{for}\;\mathrm{all}\;t\in Z\right\}$$

*is a subshift.*

**Proof.**  Clearly $S_{G}$ is σ-invariant. It remains to show that it is closed. Let $s\in CoFin{\left(N\right)}^{Z}\backslash S_{G}$. It suffices to show that *s* is an interior point. There are three cases.

(i) For all t∈Z, $s_{t}$ is not a vertex of *G*. Then ${⋃}_{v}\left\{z\in N^{Z}|z_{0}\neq v\right\}$, where *v* runs through the vertices of *G*, is an open neighborhood of *s* which does not intersect $S_{G}$.
(ii) There exists t∈Z such that $\left\{s_{t},s_{t+1}\right\}$ is not an edge of *G* but $s_{t}$ is a vertex of *G*. Then ${⋃}_{y}\left\{z\in N^{Z}|\left\{z_{t},z_{t+1}\right\}\neq \left\{s_{t},y\right\}\right\}$, where *y* runs through the vertices of *G* which fulfill the condition that $\left\{s_{t},y\right\}$ is an edge of *G*, which is an open neighborhood of *s*, does not intersect $S_{G}$.
(iii) There exists t∈Z such that $\left\{s_{t},s_{t+1}\right\}$ is not an edge of *G* but $s_{t}$ is a vertex of *G*. Then ${⋃}_{y}\left\{z\in N^{Z}|\left\{z_{t},z_{t+1}\right\}\neq \left\{y,s_{t+1}\right\}\right\}$, where *y* runs through the vertices of *G* which fulfills the condition that $\left\{y,s_{t+1}\right\}$ is an edge of *G*, which is an open neighborhood of *s*, does not intersect $S_{G}$. □

**Remark** **2.** *Let G and H be countable graphs and let m:G→H be a graph morphism. Then the induced coding $M:S_{G}\to S_{H}$ is a morphism of topological Markov chains if m is finite-to-one. This follows from Proposition 1. (See also Example 6).*

**Remark** **3** (Universal topological Markov chain). *Since there are countably many finite directed graphs, their disjoint union is a countable graph. Hence, there is a countable topological Markov chain which contains all finite topological Markov chains as closed subsystems. However, there exists a more interesting universal chain. It is well-known that there exists a countable connected directed graph U such that, for any countable directed graph G, there exists an embedding G↪U, an injection that is adjacency preserving, whose image is an induced subgraph of U. (The case of undirected graphs is discussed in [12]; the case of directed graphs, in the guise of 3-colored graphs, is discussed in [13]). The topological Markov chain $\left(S_{U},\sigma \right)$ is such that, for any topological Markov chain $S_{G}$ on a finite alphabet, there exists a closed embedding $\left(S_{G},\sigma \right)↪\left(S_{U},\sigma \right)$. To see this, observe that the universal directed graph U contains a copy of G. Let V be the finite vertex set of G. Then $V^{Z}\cap S_{U}$ is closed and nowhere dense, since V is finite, and a σ-invariant subset of $S_{U}$, since G embeds as an induced subgraph. The universal system $\left(S_{U},\sigma \right)$ contains every chain on an infinite alphabet as an open dense subsystem, since the infinite vertex set of the subgraph is open and dense in the cofinite topology.*

**Lemma** **5.** *Let G be a graph and let F be a finite induced subgraph of G. Then*

$$\exp\left({GR}_{t}\left(|W_{F}^{t}|\right)\right)=\lambda_{F}^{\max}=\lim_{t\to \infty }\sqrt[{t}]{∥F^{t}∥}$$

*for any norm ∥−∥.*

**Proof.**  Since *F* is finite, we may, without loss of generality, use the norm $∥M∥=\sum_{i,j}\left|M_{ij}\right|$. We have $|W_{F}^{t}|=\sum_{i,j}{\left(F^{t}\right)}_{ij}=∥F^{t}∥$. By Gelfand’s formula,

$$\begin{array}{ccc} \lim_{t\to \infty }\sqrt[{t}]{∥F^{t}∥}=\lim_{t\to \infty }\sqrt[{t}]{|W_{F}^{t}|} & = & \lambda_{F}^{\max}\\ \lim_{t\to \infty }\ln\left(\sqrt[{t}]{|W_{F}^{t}|}\right) & = & \ln\left(\lambda_{F}^{\max}\right)\\ \lim_{t\to \infty }\frac{1}{t}\ln\left(|W_{F}^{t}|\right) & = & \ln\left(\lambda_{F}^{\max}\right).\;□ \end{array}$$

**Proposition** **3.** *Let G be a countable graph and consider $S_{G}\subseteq N^{Z}$. Then*

$$h\left(S_{G},\sigma \right)=\sup\left\{\ln^{+}\left(\lambda_{F}^{\max}\right)|F\;\mathrm{is}\;a\;\mathrm{finite}\;\mathrm{subgraph}\;\mathrm{of}\;G\right\}.$$

**Proof.**  From Lemma 5, we know that ${GR}_{t}\left(\right|W_{F}^{t}\left|\right)=\ln\left(\lambda_{F}^{\max}\right)$, where $\lambda_{F}^{\max}$ refers to the largest eigenvalue of the subgraph induced by *F*. □

Just as a finite graph corresponds to a linear map from a finite-dimensional vector space to itself, a countable graph, an infinite {0,1}-matrix, corresponds to a linear map from an infinite-dimensional vector space to itself. In the infinite-dimensional setting the choice of topology becomes important. Let $l^{2}$ denote the Hilbert space of sequences ${\left\{x_{i}\right\}}_{i=1}^{\infty }$ such that $\sum_{i}|{x_{i}|}^{2}<\infty$ equipped with the norm ${∥x∥}_{2}=\sqrt{\sum_{i}{\left|x_{i}\right|}^{2}}$. Then the adjacency operator $G:l^{2}\to l^{2}$ is defined by

$${\left(Gx\right)}_{i}=\sum_{j=1}^{\infty }G_{ij}x_{j}.$$

We suppose that *G* is uniformly locally finite, i.e., there is a common upper bound for the number of successors of every vertex; therefore, the adjacency operator $G:l^{2}\to l^{2}$ is bounded ([14] Theorem 3.2). The spectrum of a bounded operator $B:l^{2}\to l^{2}$ is

$$\mathrm{Spec}\left(B\right)=\left\{\lambda \in C|B-\lambda I\;\mathrm{is}\;\mathrm{not}\;\mathrm{invertible}\right\},$$

and its spectral radius is the number

$$\rho \left(B\right)=\underset{\lambda }{\sup}\left\{|\lambda ||\lambda \in \mathrm{Spec}(B)\right\}.$$

**Proposition** **4.** *Let G be a uniformly locally finite directed countable graph and consider $S_{G}\subseteq N^{Z}$. Then $h\left(S_{G},\sigma \right)\leq \ln^{+}\left(\rho (G)\right)$.*

**Proof.**  We equip the space of linear maps from $l^{2}$ to itself with the operator norm ${∥-∥}_{2,2}$. We may consider a finite subgraph *F* of *G* as $F:l^{2}\to l^{2}$ by filling up with zeros. For any t∈N we have $0\leq {\left(F^{t}\right)}_{ij}\leq {\left(G^{t}\right)}_{ij}$ which implies $∥F^{t}{∥}_{2,2}\leq {∥G^{t}∥}_{2,2}$. We conclude, starting with an application of Gelfand’s formula, that

$$\begin{array}{cc} \lim_{t\to \infty }\sqrt[{t}]{∥F^{t}{∥}_{2,2}} & \leq \lim_{t\to \infty }\sqrt[{t}]{∥G^{t}{∥}_{2,2}}\\ \lambda_{F}^{\max} & \leq \rho (G)\\ \ln\left(\lambda_{F}^{\max}\right) & \leq \ln\left(\rho (G)\right)\\ \sup\ln\left(\lambda_{F}^{\max}\right) & \leq \ln\left(\rho (G)\right)\\ h(S_{G},\sigma ) & \leq \ln\left(\rho (G)\right), \end{array}$$

where we have invoked Proposition 3 in the last step. □

**Proposition** **5.** *Let G be a uniformly locally finite countable graph that is undirected, $G_{ij}=G_{ji}$, and consider $S_{G}\subseteq N^{Z}$. Then $h\left(S_{G},\sigma \right)=\ln^{+}\left(\rho (G)\right)$.*

**Proof.**  Mohar ([14] Section 4) has shown that, under the hyotheses above, $\rho \left(G\right)=\sup\left\{\lambda_{F}^{\max}|F\;\mathrm{is}\;a\;\mathrm{finite}\;\mathrm{subgraph}\;\mathrm{of}\;G\right\}$. Invoking Proposition 3 remains. □

## 6. Entropy Numbers

In this section, we show that all numbers in [0,∞] are entropies of subshifts of $N^{Z}$, in particular, entropies of topological Markov chains. This result has been obtained by Salama [15], who considered Gurevich’s compactification approach, which leads to the same entropy. In fact, Salama obtained the stronger result that, given two numbers 0≤α≤β≤∞, there exists a countable graph *G* such that $h(S_{G},\sigma )=\alpha$ and

$$h^{*}\left(S_{G}\right):=\underset{i\in N}{\sup}{GR}_{t}\left(|\{\mathrm{Words}\;\mathrm{of}\;\mathrm{length}\;t\;\mathrm{in}\;S_{G}\;\mathrm{which}\;\mathrm{begin}\;\mathrm{with}\;i\}|\right)=\beta ,$$

where the latter is an entropy-like invariant defined by Salama.

Salama’s methods are analytical, while our proof is topological. Lind [16] asked which numbers may be entropies of topological Markov chains on finite alphabets. This amounts to asking which numbers may be the spectral radii of finite directed graphs. We will need the following slight variation of a result of his.

**Lemma** **6** (Lind [16]). *There is a dense subset D⊂[1,∞) such that for every d∈D there exists a finite strongly connected directed graph G such that $\lambda_{G}^{\max}=d$.*

**Proof.**  Lind has shown that the set of Perron numbers, the real algebraic integers that dominate all their conjugates in absolute value, arise as spectral radii of positive integer matrices ([16], Theorem 1), and that the Perron numbers are dense in [1,∞) ([16], Proposition 2). By a higher block representation ([17], Section 1.2–1.7), we obtain a {0,1}-valued matrix with the same spectral radius. Since the spectral radius is realized in a strongly connected subgraph, we may choose the respective subgraph. □

The following elementary lemma can be proven by considering the Rayleigh quotient ([18] Section 1.3).

**Lemma** **7.** *Let G be a finite strongly connected graph and let S be a subgraph of G. Then $\lambda_{S}^{\max}<\lambda_{G}^{\max}$.*

**Proposition** **6** (Salama [15]). *All numbers in [0,∞] are entropies of topological Markov chains in $N^{Z}$.*

**Proof.**  The full shift has infinite entropy; see Corollary 1. The infinite one-way path has entropy zero; see Example 2. Considering the interval (0,∞) remains. Let r∈(1,∞). Consider the set *D* from Lemma 6. Since *D* is a dense subset of the linearly ordered set R, there exists a monotonically increasing sequence $\left\{d_{i}\right\}$ in *D* such that $d_{i}\to r$. Let $\left\{G_{i}\right\}$ be a sequence of finite strongly connected directed graphs such that $\lambda_{G_{i}}^{\max}=d_{i}$. The existence of such a sequence follows from Lemma 6. Take the disjoint union *G* of the topological Markov chains generated by $G_{i}$, as in Example 1. Consider the subgraph of *G* induced by a finite subset F⊂N. By Lemma 7, its spectral radius is smaller or equal to the largest $d_{i}$ such that *F* intersects the vertex set of $G_{i}$. We conclude that

$$h\left(S_{G},\sigma \right)=\underset{i}{\sup}\ln\left(\lambda_{G_{i}}^{\max}\right)=\lim_{i}\ln\left(d_{i}\right)=\ln\left(r\right).$$

Since ln((1,∞))=(0,∞), this proves the claim. □

Note that the construction in the above proof yields a nonminimal subshift. There exist minimal subshifts of $N^{Z}$ with entropy zero—for example, the subshift obtained from the infinibonacci substitution [19]. A construction of Grillenberger [20] provides a minimal subshift of $N^{Z}$ with infinite entropy.

## 7. Examples

**Example** **2.** *Consider the subshift $C\subset Z^{Z}$ defined by*

$$C:=\left\{s\in Z^{Z}|s_{t+1}=s_{t}+1\;\mathrm{for}\;\mathrm{all}\;t\in Z\right\}.$$

*We have h(C,σ)=0 since $|W_{\left\{-n,n\right\}}^{t}|\leq 2n+1$. This subshift is generated by walks along an infinite graph, the infinite one-way path. Its spectral radius is 0.*

**Example** **3.** *Consider the subshift $P\subset Z^{Z}$ defined by*

$$P:=\left\{s\in Z^{Z}||s_{t+1}-s_{t}|=1\;\mathrm{for}\;\mathrm{all}\;t\in Z\right\}.$$

*This subshift is generated by walks along an infinite graph, the infinite two-way path, whose spectral radius is 2. We have h(P,σ)=ln(2).*

**Example** **4.** *Consider the lattice $Z^{d}$ as an undirected graph. Its spectral radius is 2d. Hence, the associated topological Markov chain has entropy ln(2)+ln(d). (This generalizes Example 3).*

**Example** **5.** *Consider the undirected homogeneous q-tree. Its spectral radius is $2\sqrt{q-1}$. Hence, the associated topological Markov chain has entropy $\ln\left(2\right)+\frac{1}{2}\ln\left(q-1\right)$.*

**Example** **6.** *Consider the following two graphs.*

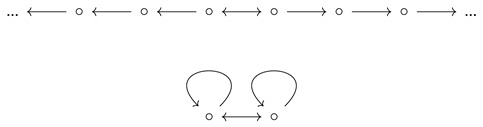

*The latter is a quotient of the former, yet the former has entropy zero, while the latter has entropy ln(2). By Proposition 1, the quotient map does not induce a continuous map between the subshifts, yet it induces an equivariant surjection whose image, the entire 2-shift, is closed.*

**Example** **7.** *Consider the following graph G on the vertex set Z∪{α,β}.*

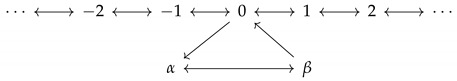

The code m:Z∪{α,β}→Z∪{*} given by

$$m\left(x\right)=\left\{\begin{array}{c} x\;\mathrm{if}\;x\in Z\\ {}*\;\mathrm{if}\;x\in \{\alpha ,\beta \} \end{array}\right.$$

induces the map $M:{\left(Z\cup \{\alpha ,\beta \}\right)}^{Z}\to {\left(Z\cup \{*\}\right)}^{Z}$ where $M{\left(s\right)}_{i}=m\left(s_{i}\right)$. By Proposition 1, $M:S_{G}\to M\left(S_{G}\right)$ is continuous. Since M is a continuous map from a compact space to a Hausdorff space, its image is closed. We conclude that M is a morphism. The image $M\left(S_{G}\right)\subset {\left(Z\cup \{*\}\right)}^{Z}$ is not generated by a graph, since whenever the symbol * appears, it must appear in a succession of evenly many *-symbols, which may not be encoded in a graph. We have $\ln\left(2\right)\leq h\left(M\left(S_{G}\right),\sigma \right)<\ln\left(2.66\right)$. This is a play on the even shift of Weiss [21].

**Example** **8.** *By considering $\bar{S_{G}}\subseteq \mathrm{Alex}{\left(N\right)}^{Z}$, one may adjoin many sequences that contain the symbol ∞. An example is the countable graph G on N which contains the edge (i,j) if and only if i≤j. Then $\bar{S_{G}}\backslash S_{G}$ equals the union of the set*

$$\left\{s\in N^{Z}|\exists z\in Z\;such\;that\;s_{t-1}\leq s_{t}<\infty \;for\;all\;t<z\;and\;s_{t}=\infty \;for\;all\;t\geq z\right\}$$

*with the constant sequence at ∞.*
